# Early Neurodevelopmental Assessments for Predicting Long-Term Outcomes in Infants at High Risk of Cerebral Palsy

**DOI:** 10.1001/jamanetworkopen.2024.13550

**Published:** 2024-05-06

**Authors:** Abdul Razak, Emily Johnston, Vathana Sackett, Marissa Clark, Margaret Charlton, Lindsay Zhou, Pramod Pharande, Courtney A. McDonald, Rod W. Hunt, Suzanne L. Miller, Atul Malhotra

**Affiliations:** 1 Department of Paediatrics, Monash University, Melbourne, Australia; 2 Monash Newborn, Monash Children’s Hospital, Melbourne, Australia; 3 The Ritchie Centre, Hudson Institute of Medical Research, Melbourne, Australia; 4 Allied Health Department, Monash Children’s Hospital, Melbourne, Australia; 5 Department of Obstetrics and Gynaecology, Monash University, Melbourne, Australia; 6 Cerebral Palsy Alliance Research, Brain and Mind Centre, University of Sydney, Sydney, Australia

## Abstract

**Importance:**

Studies suggest that early neurodevelopmental assessments are beneficial for identifying cerebral palsy, yet their effectiveness in practical scenarios and their ability to detect cognitive impairment are limited.

**Objective:**

To assess the effectiveness of early neurodevelopmental assessments in identifying cerebral palsy and cognitive and other neurodevelopmental impairments, including their severity, within a multidisciplinary clinic.

**Design, Setting, and Participants:**

This diagnostic study was conducted at Monash Children’s Hospital, Melbourne, Australia. Participants were extremely preterm infants born at less than 28 weeks’ gestation or extremely low birth weight infants less than 1000 g and term encephalopathic infants who received therapeutic hypothermia, attending the early neurodevelopmental clinic between January 2019 and July 2021. Data were analyzed from December 2023 to January 2024.

**Exposures:**

Early cerebral palsy or high risk of cerebral palsy, the absence of fidgety movements, and Hammersmith Infant Neurological Examination (HINE) scores at corrected age (CA) 3 to 4 months. Early cerebral palsy or high risk of cerebral palsy diagnosis was based on absent fidgety movements, a low HINE score (<57), and medical neurological examination.

**Main Outcome and Measures:**

The outcomes of interest were cerebral palsy, cognitive and neurodevelopmental impairments and their severity, diagnosed at 24 to 36 months’ CA.

**Results:**

A total of 116 infants (median [IQR] gestational age, 27 [25-29] weeks; 65 [56%] male) were included. Diagnosis of early cerebral palsy or high risk of cerebral palsy demonstrated a sensitivity of 92% (95% CI, 63%-99%) and specificity of 84% (95% CI, 76%-90%) for predicting cerebral palsy and 100% (95% CI, 59%-100%) sensitivity and 80% (95% CI, 72%-87%) specificity for predicting moderate to severe cerebral palsy. Additionally, the accuracy of diagnosis of early cerebral palsy or high risk of cerebral palsy was 85% (95% CI, 77%-91%) for predicting cerebral palsy and 81% (95% CI, 73%-88%) for predicting moderate to severe cerebral palsy. Similarly, the absence of fidgety movements had an 81% (95% CI, 73%-88%) accuracy in predicting cerebral palsy, and HINE scores exhibited good discriminatory power with an area under the curve of 0.88 (95% CI, 0.79-0.97) for cerebral palsy prediction. However, for cognitive impairment, the predictive accuracy was 44% (95% CI, 35%-54%) for an early cerebral palsy or high risk of cerebral palsy diagnosis and 45% (95% CI, 36%-55%) for the absence of fidgety movements. Similarly, HINE scores showed poor discriminatory power for predicting cognitive impairment, with an area under the curve of 0.62 (95% CI, 0.51-0.73).

**Conclusions and Relevance:**

In this diagnostic study of infants at high risk for cerebral palsy or other cognitive or neurodevelopmental impairment, early neurodevelopmental assessments at 3 to 4 months’ CA reliably predicted cerebral palsy and its severity at 24 to 36 months’ CA, signifying its crucial role in facilitating early intervention. However, for cognitive impairment, longer-term assessments are necessary for accurate identification.

## Key Points

**Question:**

What is the effectiveness of early neurodevelopmental assessments performed at corrected age (CA) 3 to 4 months for estimating cerebral palsy, cognitive impairment, and neurodevelopmental impairments and their severity at CA 24 to 36 months in infants at high risk of adverse neurodevelopmental outcomes?

**Findings:**

This diagnostic study including 116 infants born extremely preterm or preterm with extremely low birth weight or born full term with encephalopathy and received therapeutic hypothermia found that early neurodevelopmental assessments were effective in identifying infants with cerebral palsy and predicting its severity. However, the assessments had limited accuracy in identifying cognitive impairment and its severity, as well as in detecting any neurodevelopmental impairment and its severity.

**Meaning:**

These findings support the potential to identify cerebral palsy and its severity as early as CA 3 to 4 months through early neurodevelopmental assessments, but the role of these tests is limited in identifying cognitive and neurodevelopmental impairments.

## Teaser

This diagnostic study assess the ability of neurodevelopmental assessments conducted at corrected age 3 to 4 months to predict cerebral palsy and other neurodevelopmental impairments at corrected age 2 to 3 years in infants at high risk of adverse neurodevelopmental outcomes.

## Introduction

Prematurity and hypoxic-ischemic encephalopathy (HIE) represent critical concerns in neonatal health, posing significant risks to the well-being of newborns. Prematurity affects approximately 1 in 10 births globally,^1^ whereas HIE affects 1.6 per 1000 live births in high-income countries and 12.1 per 1000 in low- or middle-income countries.^2^ Interventions to mitigate the risk of morbidities linked to prematurity and HIE are limited,^3^ leaving affected infants at an elevated risk of neurodevelopmental complications that adversely affect motor skills and cognitive abilities. While various neurodevelopmental concerns exist, cognitive impairment and cerebral palsy stand out as primary concerns, affecting approximately 16.9% and 6.9% of very preterm infants, respectively.^4^ Early detection of these complications is recommended because prompt identification allows for timely intervention, promoting optimal motor and cognitive development in infants.^5^

Several tools for early neurodevelopmental assessments in infants are available, including the Prechtl General Movements Assessment (GMA), Hammersmith Infant Neurological Examination (HINE), and magnetic resonance imaging. While individual tools are valuable, a comprehensive approach involving these neurodevelopmental assessments, medical history, and neuroimaging is strongly recommended for early detection of cerebral palsy.^5^ However, several nuances with respect to early neurodevelopmental assessments must be addressed.

First, the existing data primarily originate from research studies, and there need to be more data from practical settings comparing the early neurodevelopmental assessments with long-term outcomes, which is essential for understanding the effectiveness in practical settings. Second, these early neurodevelopmental assessments have shown high predictability for identifying cerebral palsy, but their effectiveness in detecting cognitive and other neurodevelopmental impairments (NDI) is limited.^6^ While early neurodevelopmental assessments primarily focus on evaluating motor function and development, systematic reviews have highlighted that these assessments may be useful in identifying cognitive delay to some extent while also emphasizing the limited literature specifically addressing cognitive impairment.^6,7^ Third, there is a lack of sufficient evidence regarding the effectiveness of these tools in evaluating other NDI commonly used in clinical trials, especially those incorporating a sensory component alongside cognitive and motor components. To address these knowledge gaps, our study was designed to assess the broader ability of early neurodevelopmental assessments to predict cerebral palsy, cognitive impairment, and other NDI, including their severity, within a practical setting, at 24 to 36 months’ corrected age (CA).

## Methods

This diagnostic study was approved by the Monash Health Human Research Ethics Committee as a quality assurance project, and parental consent was not required because it was an observational study and the exposure was considered within routine care. This study is reported following the Standards for Reporting of Diagnostic Accuracy (STARD) reporting guideline.

### Design, Setting, and Participants

This diagnostic study assessed test accuracy by comparing observational data from our multidisciplinary, early neurodevelopmental clinic at CA 3 to 4 months with long-term neurodevelopmental outcomes at CA 24 to 36 months. This study included children born between January 1, 2019, and July 30, 2021. We chose this time frame to allow for neurodevelopmental outcome assessments, with the end point for these assessments set at 36 months. Infants considered at high risk of cerebral palsy or other NDI were recruited from the early neurodevelopmental clinic^8^ at Monash Children’s Hospital, Melbourne, Australia, a perinatal and surgical tertiary neonatal unit providing care to preterm and term infants in Victoria, Australia. These infants were born extremely preterm (born before 28 weeks) or had extremely low birth weight (<1000 g) or had moderate to severe HIE who received therapeutic hypothermia.

### Index Test

Skilled physiotherapists, occupational therapists, and neonatologists trained in GMA conducted early neurodevelopmental assessments at CA 3 to 4 months, using the Prechtl GMA^9^ and the HINE.^10^ The primary predictor was early diagnosis of cerebral palsy or a high risk of cerebral palsy. Additional predictors included the absence of fidgety movements, HINE score, and early diagnosis of neurodevelopmental impairment (NDI). Early cerebral palsy or high risk of cerebral palsy was diagnosed based on absent fidgety movements, low HINE score (<57), and a medical neurological examination involving motor function, muscle tone, reflexes, and coordination. The diagnosis of early NDI included any anomalies found during initial assessments. These could be isolated anomalies, like absent fidgety movements, a low HINE score (<57), or isolated issues noted during medical neurological examinations. It could also encompass multiple anomalies, such as a combination of 2 or more anomalies or a diagnosis of early cerebral palsy or high risk of cerebral palsy.

### Reference Standard

Skilled neuropsychologists certified in Bayley Scales of Infant & Toddler Development (BSID) assessments and experienced developmental pediatricians conducted long-term neurodevelopmental evaluations between CA 24 and 36 months. The evaluations encompassed medical neurodevelopmental examination, BSID assessments (III or IV edition),^11^ and assessments for blindness and deafness as part of the Australian and New Zealand Neonatal Network (ANZNN)^12^ data collection. Our primary outcomes were any grade of cerebral palsy diagnosed on medical neurological examination and moderate to severe cerebral palsy, defined as cerebral palsy with grade II or above based on Gross Motor Function Classification System. We assessed 4 secondary outcomes: cognitive impairment, moderate to severe cognitive impairment, any NDI, and moderate to severe NDI. Cognitive impairment was defined as cognitive score less than 1 SD from the mean based on BSID III or IV. Moderate to severe cognitive impairment was defined as cognitive score less than 2 SD from the mean based on BSID III or IV. Any NDI was defined as the presence of any the following: cerebral palsy; motor, language, or cognitive impairment (score <1 SD on BSID); blindness (vision <6/60 in the better eye), or deafness (need for hearing aid). Moderate to severe NDI was defined as the presence of any following: moderate to severe cerebral palsy, moderate to severe motor, language, or cognitive impairment (score <2 SD on BSID); blindness, or deafness.

### Data Collection

The early neurodevelopmental clinic at Monash Children’s Hospital prospectively collected neurodevelopmental data at CA 3 to 4 months, while demographic and long-term data were obtained from the ANZNN, a collaborative network encompassing all 29 neonatal intensive care units in Australia and New Zealand. Monash Children’s Hospital contributes data to ANZNN, which systematically gathers information for preterm infants born at less than 32 weeks’ gestational age admitted to any participating neonatal intensive care unit. Specifically, ANZNN collects neurodevelopmental data for infants at high risk of adverse neurodevelopmental outcomes up to ages 2 to 3 years, including infants born before 28 weeks’ gestation or weighing less than 1000 g at birth, as well as infants with moderate to severe HIE.

### Statistical Analyses

Diagnostic test accuracy values, along with corresponding 95% CIs, were calculated for early cerebral palsy or high risk of cerebral palsy, early NDI, and absent fidgety movements using MedCalc statistical software version 19.2.6 (MedCalc Software). Receiver operating characteristic curve analysis was conducted to obtain the area under the curve (AUC) and 95% CI for the HINE score, using SPSS software, 2021 release (IBM). Other analyses, including summarizing demographic variables (presented as the median and IQR due to nonnormal distribution) and predictive probability of cerebral palsy, were performed using Stata software version 17.0 (StataCorp). Data were analyzed from December 2023 to January 2024.

## Results

A total of 116 infants (median [IQR] gestational age, 27 [25-29] weeks; 65 [56%] male) were included in the study, comprising 100 preterm infants and 16 term infants. Further details about these infants, including antenatal information, delivery details, resuscitation, postnatal morbidities, and outcomes, are reported in the eTable in Supplement 1. Early neurodevelopmental assessments were conducted at a median (IQR) of 13 (13-14) weeks’ CA, whereas long-term neurodevelopmental outcome evaluations were performed at a median (IQR) of 33 (30-35) months’ CA. The prevalence of cerebral palsy was 11% overall (13 of 116 infants), with 8% occurring among preterm infants (8 of 100 infants) and 31% among term infants with HIE (5 of 16 infants). Furthermore, the prevalence of cognitive impairment was 64% overall (70 of 109 infants), with 60% observed among preterm infants (56 of 93 infants) and 87% among term infants with HIE (14 of 16 infants). Three infants had hearing loss requiring amplification, and 1 infant had blindness.

### Primary Outcome

**Figure 1.**
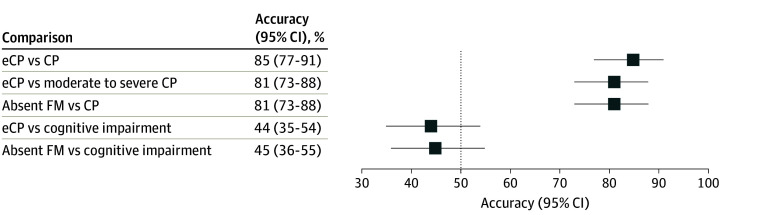
Accuracy of Early Neurodevelopmental Assessments and Absence of Fidgety Movements at Corrected Age 3 to 4 Months Compared With Neurodevelopmental Outcomes Assessed at Corrected Age 24 to 36 Months CP indicates cerebral palsy; eCP, early diagnosis of CP or high risk of CP; FM, fidgety movement.

**Figure 2.**
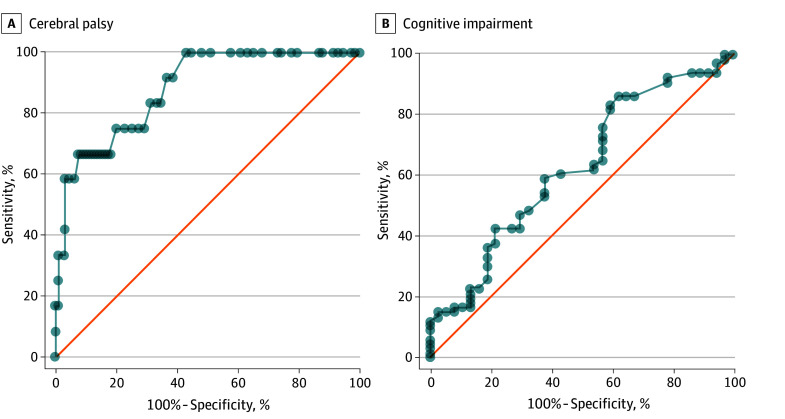
Receiver Operating Characteristic Curve for Hammersmith Infant Neurological Examination Scores in Predicting Cerebral Palsy and Cognitive Impairment The area under the curve was 0.88 (95% CI, 0.79-0.97) for cerebral palsy (A) and 0.62 (95% CI, 0.51-0.73) for cognitive impairment (B).

The early cerebral palsy or high risk of cerebral palsy diagnosis demonstrated an accuracy of 85% (95% CI, 77% to 91%), correctly identifying 99 of 116 infants evaluated for cerebral palsy (Table 1 and Figure 1). Among 13 infants diagnosed with cerebral palsy, the early cerebral palsy or high risk of cerebral palsy diagnosis showed a sensitivity of 92% (95% CI, 63% to 99%) by correctly identifying 12 infants. Notably, it achieved a 100% (95% CI, 59% to 100%) sensitivity (7 of 7 infants) in identifying all patients with moderate to severe cerebral palsy. Furthermore, the absence of fidgety movements exhibited a comparable accuracy of 81% (95% CI, 73% to 88%), and the HINE score displayed good discriminatory power, with an AUC of 0.88 (95% CI, 0.79 to 0.97) for predicting cerebral palsy (Figure 2). On the other hand, the early NDI predictor, while correctly identifying all infants with cerebral palsy, also yielded false positives, resulting in a lower accuracy of 48% (95% CI, 38% to 57%).

**Table 1.** Diagnostic Test Accuracy Values of Early Neurodevelopmental Outcomes for Predicting Cerebral Palsy

Finding	% (95% CI)					Positive likelihood ratio (95% CI)	Negative likelihood ratio (95% CI)	AUC (95% CI)
	Accuracy	Sensitivity	Specificity	PPV	NPV			
**Cerebral palsy**								
eCP	85 (77-91)	92 (63-99)	84 (76-90)	42 (31-54)	98 (92-99)	5.95 (3.69-9.57)	0.09 (0.01-0.6)	NA
Absent fidgety movements	81 (73-88)	76 (46-94)	82 (73-88)	35 (24-48)	96 (90-98)	4.27 (2.56-7.14)	0.28 (0.10-0.76)	NA
eNDI	48 (38-57)	100 (75-100)	41 (32-58)	17 (15-23)	100 (91-100)	1.72 (1.46-2.02)	0	NA
HINE score	NA	NA	NA	NA	NA	NA	NA	0.88 (0.79-0.97)
**Moderate to severe cerebral palsy**								
eCP	81 (73-88)	100 (59-100)	80 (72-87)	25 (18-32)	100 (95-100)	5.19 (3.53-7.62)	0	NA
Absent fidgety movements	81 (73-88)	100 (59-100)	80 (71-82)	25 (18-32)	100 (95-100)	1.65 (1.42-1.92)	0	NA
eNDI	43 (33-52)	100 (59-100)	39 (30-49)	9 (8-10)	100 (91-100)	5.05 (3.44-7.40)	0	NA
HINE score	NA	NA	NA	NA	NA	NA	NA	0.88 (0.78-0.97)

Abbreviations: AUC, area under the curve; eCP, early diagnosis of cerebral palsy or high risk of cerebral palsy; eNDI, early diagnosis of neurodevelopmental impairment; HINE, Hammersmith Infant Neurological Examination; NA, not applicable; NPV, negative predictive value; PPV, positive predictive value.

**Figure 3.**
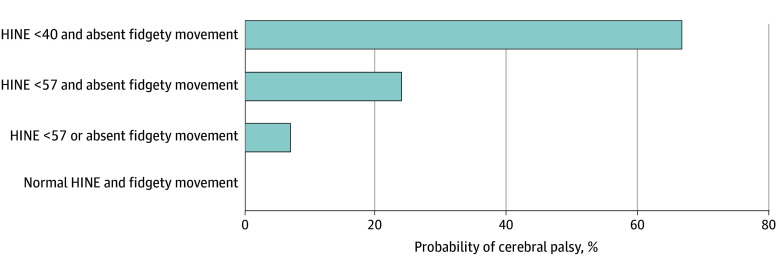
Probability of Cerebral Palsy by Anomalies Identified on the Early Neurodevelopmental Assessments HINE indicates Hammersmith Infant Neurological Examination.

The probability of cerebral palsy in infants was calculated depending on the anomalies noted on the early assessments, with none of the infants developing cerebral palsy having a HINE score within reference range (>57) and fidgety movements. The probability was noted at 7% if an infant had either a low HINE score or absent fidgety movements. If both were present (low HINE score and absent fidgety movements), the probability was 24%, which was not significantly different compared with infants with either a low HINE score or absent fidgety movements alone (absolute difference, 17% [95% CI, −2% to 36%]). However, infants with a very low HINE score (<40) and absent fidgety movements had a significantly higher predicted probability of 67%, in contrast with infants with either a low HINE score or absent fidgety movements alone (absolute difference, 60% [95% CI, 21% to 98%]) (Figure 3).

### Secondary Outcomes

#### Cognitive Impairment

The accuracy of various early neurodevelopmental assessments was notably limited, ranging from 44% to 60%, in predicting cognitive impairment and its severity (Table 2 and Figure 1). Although the specificity of certain assessments showed modest values, ranging between 76% and 80%, the sensitivity and positive and negative predictive values demonstrated suboptimal performance. Additionally, the HINE score displayed limited discriminatory power in predicting cognitive impairment, reflected by an AUC of 0.62 (95% CI, 0.51 to 0.73) (Figure 2).

**Table 2.** Diagnostic Test Accuracy Values of Early Neurodevelopmental Outcomes for Predicting Cognitive Impairment and NDI

Finding	% (95% CI)					Positive likelihood ratio (95% CI)	Negative likelihood ratio (95% CI)	AUC (95% CI)
	Accuracy	Sensitivity	Specificity	PPV	NPV			
**Cognitive impairment**								
eCP	44 (35-54)	24 (14-36)	80 (64-90)	68 (50-81)	37 (32-42)	1.21 (0.58-2.56)	0.95 (0.77-1.16)	NA
Absent fidgety movements	45 (36-55)	26 (16-38)	81 (65-92)	72 (54-84)	37 (33-42)	1.42 (0.65-2.08)	0.91 (0.74-1.11)	NA
eNDI	60 (51-70)	67 (58-77)	50 (33-66)	63 (53-72)	46 (35-57)	1.34 (0.95-1.91)	0.66 (0.42-1.04)	NA
HINE score	NA	NA	NA	NA	NA	NA	NA	0.62 (0.51-0.73)
**Moderate to severe cognitive impairment**								
eCP	58 (48-62)	21 (9-38)	76 (65-85)	33 (24-43)	65 (60-70)	0.93 (0.44-1.95)	1.02 (0.83-1.26)	NA
Absent fidgety movements	59 (49-69)	25 (12-42)	77 (66-84)	36 (21-53)	67 (61-71)	1.11 (0.54-2.26)	0.97 (0.77-1.21)	NA
eNDI	52 (42-62)	70 (53-84)	43 (32-55)	38 (32-45)	74 (62-83)	1.25 (0.93-1.67)	0.68 (0.39-1.67)	NA
HINE score	NA	NA	NA	NA	NA	NA	NA	0.60 (0.49-0.72)
**NDI**								
eCP	31 (23-40)	23 (15-33)	70 (45-88)	78 (63-88)	16 (12-20)	0.78 (0.36-1.67)	1.09 (0.80-1.49)	NA
Absent fidgety movements	34 (25-43)	25 (16-35)	75 (50-91)	82 (66-91)	18 (14-22)	1.01 (0.44-2.34)	1.00 (0.75-1.32)	NA
eNDI	58 (49-67)	62 (52-72)	40 (19-63)	83 (76-87)	18 (11-29)	1.05 (0.71-1.55)	0.93 (0.51-1.69)	NA
HINE score	NA	NA	NA	NA	NA	NA	NA	0.51 (0.37-0.66)
**Moderate to severe NDI**								
eCP	58 (48-67)	32 (20-45)	83 (71-92)	66 (49-80)	55 (49-60)	2.00 (0.98-4.07)	0.81 (0.65-1.00)	NA
Absent fidgety movements	57 (47-67)	32 (20-46)	83 (70-92)	66 (49-80)	54 (49-60)	1.96 (0.97-3.98)	0.81 (0.65-1.01)	NA
eNDI	59 (50-68)	71 (57-82)	48 (34-61)	57 (50-65)	62 (50-73)	1.38 (1.02-1.87)	0.59 (0.36-0.9)	NA
HINE score	NA	NA	NA	NA	NA	NA	NA	0.60 (0.49-0.71)

Abbreviations: AUC, area under the curve; eCP, early diagnosis of cerebral palsy or high risk of cerebral palsy; eNDI, early diagnosis of neurodevelopmental impairment; HINE, Hammersmith infant neurological examination; NDI, neurodevelopmental impairment; NPV, negative predictive value; PPV, positive predictive value.

#### NDI

Similar to cognitive impairment, the predictive accuracy of various early neurodevelopmental assessments was notably low, ranging from 31% to 59%, when predicting NDI and its severity (Table 2). Moreover, the sensitivity and negative predictive values showed suboptimal performance across the different assessments, while the positive predictive value and specificity were modest in predicting NDI and its severity, respectively.

## Discussion

This diagnostic study, leveraging data from infants considered at high risk of adverse neurodevelopmental outcomes, assessed the effectiveness of early neurodevelopmental assessments at CA 3 to 4 months in identifying cerebral palsy, cognitive impairment, and other NDI and their severity at CA 24 to 36 months. Early assessments, including early cerebral palsy or high risk of cerebral palsy, absent fidgety movements, and HINE scores, exhibited high accuracy in identifying cerebral palsy. Moreover, these assessments achieved a sensitivity and negative predictive value of 100% for detecting moderate to severe cerebral palsy. Furthermore, the study found an increase in the predicted probability of cerebral palsy with the number and severity of anomalies identified in early neurodevelopmental assessments. With respect to predicting cognitive and neurodevelopmental impairment and their severity, we found limited accuracy, sensitivity, and negative predictive value of early neurodevelopmental assessments.

While current research using observational data offers insights into the feasibility of early neurodevelopmental assessments for diagnosing cerebral palsy at an early stage,^8,13,14,15,16,17,18,19^ there exists a notable gap in studies directly comparing early data with long-term outcomes. Observational data studies are crucial, as they offer insights into the effectiveness of assessments in contextual settings. Although systematic reviews of research studies have consistently shown high accuracy in early neurodevelopmental assessments for identifying cerebral palsy,^20,21,22^ the direct applicability of these findings in clinical settings is limited. Some studies using clinical data have found lower sensitivities or accuracies in predicting cerebral palsy,^23,24,25^ while others have found higher accuracy.^26^ This discrepancy is likely influenced by various factors, including the characteristics of the studied population,^25^ the prevalence of cerebral palsy or NDI,^27^ and the level of training in the assessments.^28,29^ Additionally, some researchers argue that continued follow-up assessments may enhance accuracy compared with 1-time assessments.^30^ Nevertheless, our findings support the notion that these assessments maintain high accuracy in practical settings, aligning with both previous research^20,21,22^ and clinical studies.^26^

Using our study results in a simulated scenario of 100 infants with high risk of cerebral palsy or NDI, with an observed 11% incidence of cerebral palsy and a 6% incidence of moderate to severe cerebral palsy, the data suggest that standardized assessments with a finding of early cerebral palsy or high risk of cerebral palsy at CA 3 to 4 months would identify 10 of 11 infants with cerebral palsy and all infants with moderate to severe cerebral palsy. However, this diagnosis would also falsely identify 15 infants as having cerebral palsy. In contrast, if the early cerebral palsy or high risk of cerebral palsy diagnosis is used to identify NDI, considering a 64% incidence of NDI from this study, this diagnosis would only detect 15 of 64 infants with NDI while incorrectly identifying 20 infants with NDI. Overall, these data confirm the high sensitivity and accuracy of early standardized assessments in detecting cerebral palsy and gauging its severity, aligning with prior research^20,21,22^; however, our findings underscore the restricted effectiveness of these assessments in identifying cognitive impairment and NDI and their severity.

Ideally, a test should be able to identify all patients with the disease, but a highly sensitive test often comes with an increased false positive rate. This phenomenon was observed in our study, where the early cerebral palsy or high risk of cerebral palsy diagnosis exhibited high sensitivity but had a 15% false positivity rate. When assessing this false positivity rate, it is worth considering that it might have been influenced by the fact that infants diagnosed with early cerebral palsy or high risk of cerebral palsy underwent early intervention, potentially positively impacting their development.^31^ Such early interventions could have altered the developmental trajectory of these infants, particularly for those with milder symptoms, ultimately preventing them from receiving a final diagnosis of cerebral palsy. In Australia, infants diagnosed with cerebral palsy and other disabilities have access to national disability insurance scheme funding provided by both the Commonwealth and state governments, ensuring equitable support without disparities.

Early neurodevelopmental assessments are customarily designed to focus on motor evaluation and do not inherently encompass the assessment of cognitive domains. While there is a possibility that these assessments may indirectly offer insights into cognitive domains,^5,6,17^ given that disorders affecting cognition may also impact motor function, our study’s findings do not demonstrate their utility in assessing cognitive impairments. Furthermore, their utility in addressing broader NDI is constrained, as this outcome is comprehensive and involves cognitive impairment within its definition.

Our study has several notable strengths. Unlike previous investigations reliant on research outputs, our study draws on observational data gathered directly from clinical settings. This distinctive approach lends credibility to our results, reflecting the practical application of early neurodevelopmental assessments. Another strength of our study is the meticulous prospective collection of data, ensuring the acquisition of reliable and valid information while minimizing the risk of recall bias. Furthermore, the assessors of long-term outcomes used standardized objective measures during assessments, which further reduces the risk of bias in the assessment process. Additionally, the comprehensive nature of our comparisons contributes to our study’s depth, thereby enhancing the robustness of our findings.

### Limitations

It is important to acknowledge some limitations. While our study comprehensively compared early neurodevelopmental assessments with long-term outcomes in a clinical setting, the study population was relatively small, as evidenced by wide CIs in some diagnostic test accuracy measures. Additionally, the data were sourced from a single center, which may restrict the generalizability of our findings. Furthermore, the inclusion of infants with high risk, particularly with most infants being born extremely preterm, means that our findings may not be directly applicable to a more diverse range of infant populations.

## Conclusions

In this diagnostic study of infants at high risk of cerebral palsy and NDI, our findings affirm the effectiveness of early standardized assessments in detecting cerebral palsy and determining its severity as early as CA 3 to 4 months, even in clinical settings. This emphasizes the crucial role of integrating these assessments into clinical practice, enabling early interventions with the potential to influence the progression of the disease. Furthermore, our findings indicate that these assessments have limited utility in detecting cognitive impairment and other NDI, emphasizing the ongoing necessity for longer-term assessments to accurately identify impairments beyond cerebral palsy.

## Notes

Supplement 1. **eTable.** Characteristics, Morbidities, and Outcomes of Infants at High Risk of Cerebral Palsy

Supplement 2.
Data Sharing Statement

## References

[1] Ohuma EO, Moller AB, Bradley E, . National, regional, and global estimates of preterm birth in 2020, with trends from 2010: a systematic analysis. Lancet. 2023;402(10409):1261-1271. doi:10.1016/S0140-6736(23)00878-4 37805217

[2] Lee AC, Kozuki N, Blencowe H, . Intrapartum-related neonatal encephalopathy incidence and impairment at regional and global levels for 2010 with trends from 1990. Pediatr Res. 2013;74(suppl 1):50-72. doi:10.1038/pr.2013.206 24366463 PMC3873711

[3] Razak A, Patel W, Durrani NUR, Pullattayil AK. Interventions to reduce severe brain injury risk in preterm neonates: a systematic review and meta-analysis. JAMA Netw Open. 2023;6(4):e237473. doi:10.1001/jamanetworkopen.2023.7473 37052920 PMC10102877

[4] Pascal A, Govaert P, Oostra A, Naulaers G, Ortibus E, Van den Broeck C. Neurodevelopmental outcome in very preterm and very-low-birthweight infants born over the past decade: a meta-analytic review. Dev Med Child Neurol. 2018;60(4):342-355. doi:10.1111/dmcn.13675 29350401

[5] Novak I, Morgan C, Adde L, . Early, accurate diagnosis and early intervention in cerebral palsy: advances in diagnosis and treatment. JAMA Pediatr. 2017;171(9):897-907. doi:10.1001/jamapediatrics.2017.1689 28715518 PMC9641643

[6] Einspieler C, Bos AF, Libertus ME, Marschik PB. The General Movement Assessment helps us to identify preterm infants at risk for cognitive dysfunction. Front Psychol. 2016;7:406. doi:10.3389/fpsyg.2016.00406 27047429 PMC4801883

[7] Caesar R, Colditz PB, Cioni G, Boyd RN. Clinical tools used in young infants born very preterm to predict motor and cognitive delay (not cerebral palsy): a systematic review. Dev Med Child Neurol. 2021;63(4):387-395. doi:10.1111/dmcn.14730 33185285

[8] King AR, Machipisa C, Finlayson F, Fahey MC, Novak I, Malhotra A. Early detection of cerebral palsy in high-risk infants: translation of evidence into practice in an Australian hospital. J Paediatr Child Health. 2021;57(2):246-250. doi:10.1111/jpc.15191 32940939

[9] Einspieler C, Prechtl HF. Prechtl’s assessment of general movements: a diagnostic tool for the functional assessment of the young nervous system. Ment Retard Dev Disabil Res Rev. 2005;11(1):61-67. doi:10.1002/mrdd.20051 15856440

[10] Romeo DM, Ricci D, Brogna C, Mercuri E. Use of the Hammersmith Infant Neurological Examination in infants with cerebral palsy: a critical review of the literature. Dev Med Child Neurol. 2016;58(3):240-245. doi:10.1111/dmcn.12876 26306473

[11] Bayley NA. Bayley Scales of Infant and Toddler Development. 4th ed. Pearson; 2019.

[12] Australian and NewZealand Neonatal Network. https://www.anznn.net/dataresources/datadictionaries. Published 2018. Accessed.

[13] Byrne R, Noritz G, Maitre NL; NCH Early Developmental Group. Implementation of early diagnosis and intervention guidelines for cerebral palsy in a high-risk infant follow-up clinic. Pediatr Neurol. 2017;76:66-71. doi:10.1016/j.pediatrneurol.2017.08.002 28982529

[14] Davidson SA, Ward R, Elliott C, ; Group EI at-risk CP Team. From guidelines to practice: a retrospective clinical cohort study investigating implementation of the early detection guidelines for cerebral palsy in a state-wide early intervention service. BMJ Open. 2022;12(11):e063296. doi:10.1136/bmjopen-2022-063296 36428013 PMC9703326

[15] Huang HB, Watt MJ, Hicks M, . A family-centered, multidisciplinary clinic for early diagnosis of neurodevelopmental impairment and cerebral palsy in China—a pilot observation. Front Pediatr. 2022;10:840190. doi:10.3389/fped.2022.840190 35372170 PMC8968569

[16] Maitre NL, Burton VJ, Duncan AF, . Network implementation of guideline for early detection decreases age at cerebral palsy diagnosis. Pediatrics. 2020;145(5):e20192126. doi:10.1542/peds.2019-2126 32269135 PMC7193973

[17] Martínez Moreno M, Macias Merlo L. Early detection and intervention in cerebral palsy: from knowledge to action. Dev Med Child Neurol. 2022;64(5):529. doi:10.1111/dmcn.15178 35383896

[18] Te Velde A, Tantsis E, Novak I, . Age of diagnosis, fidelity and acceptability of an early diagnosis clinic for cerebral palsy: a single site implementation study. Brain Sci. 2021;11(8):1074. doi:10.3390/brainsci11081074 34439692 PMC8391606

[19] Zhong B, Tan K, Razak A, . Early neurodevelopmental outcomes of extreme preterm infants exposed to paracetamol: a retrospective cohort study. Pediatr Res. 2023;94(5):1714-1719. doi:10.1038/s41390-023-02649-4 37198403 PMC10189702

[20] Bosanquet M, Copeland L, Ware R, Boyd R. A systematic review of tests to predict cerebral palsy in young children. Dev Med Child Neurol. 2013;55(5):418-426. doi:10.1111/dmcn.12140 23574478

[21] Darsaklis V, Snider LM, Majnemer A, Mazer B. Predictive validity of Prechtl’s Method on the Qualitative Assessment of General Movements: a systematic review of the evidence. Dev Med Child Neurol. 2011;53(10):896-906. doi:10.1111/j.1469-8749.2011.04017.x 21679361

[22] Kwong AKL, Fitzgerald TL, Doyle LW, Cheong JLY, Spittle AJ. Predictive validity of spontaneous early infant movement for later cerebral palsy: a systematic review. Dev Med Child Neurol. 2018;60(5):480-489. doi:10.1111/dmcn.13697 29468662

[23] Constantinou JC, Adamson-Macedo EN, Mirmiran M, Fleisher BE. Movement, imaging and neurobehavioral assessment as predictors of cerebral palsy in preterm infants. J Perinatol. 2007;27(4):225-229. doi:10.1038/sj.jp.7211664 17304207

[24] Datta AN, Furrer MA, Bernhardt I, ; GM Group. Fidgety movements in infants born very preterm: predictive value for cerebral palsy in a clinical multicentre setting. Dev Med Child Neurol. 2017;59(6):618-624. doi:10.1111/dmcn.13386 28102574

[25] Støen R, Boswell L, de Regnier RA, . The predictive accuracy of the General Movement Assessment for Cerebral Palsy: a prospective, observational study of high-risk infants in a clinical follow-up setting. J Clin Med. 2019;8(11):1790. doi:10.3390/jcm8111790 31717717 PMC6912231

[26] Morgan C, Crowle C, Goyen TA, . Sensitivity and specificity of General Movements Assessment for diagnostic accuracy of detecting cerebral palsy early in an Australian context. J Paediatr Child Health. 2016;52(1):54-59. doi:10.1111/jpc.12995 26289780

[27] Prechtl HF, Einspieler C, Cioni G, Bos AF, Ferrari F, Sontheimer D. An early marker for neurological deficits after perinatal brain lesions. Lancet. 1997;349(9062):1361-1363. doi:10.1016/S0140-6736(96)10182-3 9149699

[28] Ferrari F, Cioni G, Einspieler C, . Cramped synchronized general movements in preterm infants as an early marker for cerebral palsy. Arch Pediatr Adolesc Med. 2002;156(5):460-467. doi:10.1001/archpedi.156.5.460 11980551

[29] Spittle AJ, Spencer-Smith MM, Cheong JL, . General movements in very preterm children and neurodevelopment at 2 and 4 years. Pediatrics. 2013;132(2):e452-e458. doi:10.1542/peds.2013-0177 23878041

[30] Maitre NL, Slaughter JC, Aschner JL. Early prediction of cerebral palsy after neonatal intensive care using motor development trajectories in infancy. Early Hum Dev. 2013;89(10):781-786. doi:10.1016/j.earlhumdev.2013.06.004 23856349 PMC3759619

[31] Blauw-Hospers CH, Hadders-Algra M. A systematic review of the effects of early intervention on motor development. Dev Med Child Neurol. 2005;47(6):421-432. doi:10.1111/j.1469-8749.2005.tb01165.x 15934492

