# Supplementary material for: Early Neurodevelopmental Assessments for Predicting Long-Term Outcomes in Infants at High Risk of Cerebral Palsy
Source: JAMA Netw Open. 2024 May 6;7(5):e2413550. doi: 10.1001/jamanetworkopen.2024.13550 (PMC11074812; doi:10.1001/jamanetworkopen.2024.13550)
Supplement: Supplement 1. — eTable. Characteristics, Morbidities, and Outcomes of Infants at High Risk of Cerebral Palsy [file jamanetwopen-e2413550-s001.pdf]

Supplemental Online Content

Razak A, Johnston E, Sackett V, et al. Early neurodevelopmental assessments for predicting long-term outcomes in infants at high risk of cerebral palsy. *JAMA Netw Open*. 2024;7(5):e2413550. doi:10.1001/jamanetworkopen.2024.13550

eTable. Characteristics, Morbidities, and Outcomes of Infants at High Risk of Cerebral Palsy

This supplemental material has been provided by the authors to give readers additional information about their work.

© 2024 [First author last name and initials or group name] et al. *[Journal name].*

e-table 1: Characteristics, Morbidities, and Outcomes of Infants at High Risk of Cerebral Palsy

Antenatal	
Gestational age (median (interquartile range))	27 (25, 29)
Birthweight (median (interquartile range))	834 (687, 1018)
Male sex (% (n/N))	56 (65/116)
Multiple pregnancies (% (n/N))	16 (19/116)
Antenatal corticosteroids (% (n/N))*	
• Partial	30 (30/97)
• Complete	62 (61/97)
Antenatal magneisum sulfate (% (n/N))*	81 (79/97)
Chorioamnionitis (% (n/N))	29 (32/108)
Cesarean section (% (n/N))	68 (79/116)
Perinatal	
APGAR score at 1 min (median (interquartile range))	5 (3, 7)
APGAR score at 5 min (median (interquartile range))	7 (6, 8)
Delayed cord clamping (% (n/N))	32 (30/91)
Positive pressure ventilation at birth (% (n/N))	83 (97/116)
Intubation at birth (% (n/N))	31 (36/116)
Chest compressions at birth (% (n/N))	8 (10/116)
Adrenaline at birth (% (n/N))	2 (3/116)
Postnatal	
Thrombocytopenia (% (n/N))	22 (26/116)
Coagulopathy (% (n/N))	59 (38/64)
Inotrope therapy (% (n/N))	28 (33/116)
Blood products (% (n/N))	61 (71/116)
Postnatal corticosteroids therapy (% (n/N))*	29 (29/100)
Culture-positive sepsis (% (n/N))	42 (49/116)
Intraventricular hemorrhage (% (n/N))	41 (48/116)
Periventricular leukomalacia (% (n/N))	19 (23/116)
Patent ductus arteriosus requiring treatment (% (n/N))	36 (42/116)
Retinopathy of prematurity requiring treatment (% (n/N))*	16 (16/100)
Necrotizing enterocolitis requiring surgery (% (n/N))	3 (4/116)
Chronic lung disease (% (n/N))*	61 (61/100)
Home oxygen therapy (% (n/N))*	24 (24/100)

Note: *Information captured only for preterm infants

© 2024 [First author last name and initials or group name] et al. *[Journal name].*
