# Supplementary material for: Early Neurodevelopmental Assessments for Predicting Long-Term Outcomes in Infants at High Risk of Cerebral Palsy
Source: JAMA Netw Open. 2024 May 6;7(5):e2413550. doi: 10.1001/jamanetworkopen.2024.13550 (PMC11074812; doi:10.1001/jamanetworkopen.2024.13550)
Supplement: Supplement 2. — Data Sharing Statement [file jamanetwopen-e2413550-s002.pdf]

Data Sharing Statement

Razak. Early Neurodevelopmental Assessments for Predicting Long-Term Outcomes in Infants at High Risk of Cerebral Palsy. *JAMA Netw Open*. Published May 06, 2024. doi:10.1001/jamanetworkopen.2024.13550

Data
Data available: No
